# Evaluation of large-group lectures in medicine – development of the SETMED-L (Student Evaluation of Teaching in MEDical Lectures) questionnaire

**DOI:** 10.1186/s12909-017-0970-8

**Published:** 2017-08-18

**Authors:** Tjark Müller, Diego Montano, Herbert Poinstingl, Katharina Dreiling, Sarah Schiekirka-Schwake, Sven Anders, Tobias Raupach, Nicole von Steinbüchel

**Affiliations:** 1 Department of Legal Medicine, University Medical Centre Hamburg-Eppendorf, Butenfeld 34, D-22529 Hamburg, Germany; 2 Institute of Medical Psychology and Medical Sociology, Georg-August-University Göttingen, Waldweg 37, D-37075 Göttingen, Germany; 3 Department of Cardiology and Pneumology, University Medical Centre Göttingen, Robert-Koch-Straße 40, D-37075 Göttingen, Germany; 4 Division of Medical Education Research and Curriculum Development, Göttingen University Medical Centre, Robert-Koch-Straße 40, D-37075 Göttingen, Germany

**Keywords:** Evaluation, Lecture, Medical education, Psychometrics, Questionnaire, SETMED-L

## Abstract

**Background:**

The seven categories of the Stanford Faculty Development Program (SFDP) represent a framework for planning and assessing medical teaching. Nevertheless, so far there is no specific evaluation tool for large-group lectures that is based on these categories. This paper reports the development and psychometric validation of a short German evaluation tool for large-group lectures in medical education (SETMED-L: ‘Student Evaluation of Teaching in MEDical Lectures’) based on the SFDP-categories.

**Methods:**

Data were collected at two German medical schools. In Study 1, a full information factor analysis of the new 14-item questionnaire was performed. In Study 2, following cognitive debriefings and adjustments, a confirmatory factor analysis was performed. The model was tested for invariance across medical schools and student gender. Convergent validity was assessed by comparison with results of the FEVOR questionnaire.

**Results:**

Study 1 (*n* = 922) yielded a three-factor solution with one major (10 items) and two minor factors (2 items each). In Study 2 (*n* = 2740), this factor structure was confirmed. Scale reliability ranged between α = 0.71 and α = 0.88. Measurement invariance was given across student gender but not across medical schools. Convergent validity in the subsample tested (*n* = 246) yielded acceptable results.

**Conclusion:**

The SETMED-L showed satisfactory to very good psychometric characteristics. The main advantages are its short yet comprehensive form, the integration of SFDP-categories and its focus on medical education.

**Electronic supplementary material:**

The online version of this article (doi:10.1186/s12909-017-0970-8) contains supplementary material, which is available to authorized users.

## Background

Evaluation is essential for high-quality education. Not only can it be used for curriculum development, it can also give instructors feedback on their performance and shed light on possible shortcomings [1]. However to provide useful information, evaluation tools need to address particularities of their subjects.

Medical education differs from other higher education curricula. The curriculum structure requires students to take predefined courses rather than making individual choices. Some teaching formats are unique to medical education, like in-patient or bedside teaching. Specific evaluation tools need to reflect aspects that are specific to medical education. Large group lectures might appear to be comparable to those in other higher education curriculums and thus important differences might be overlooked.

In medical education it is more common for course series that sessions are not all held by a single instructor but by different lecturers [2]. This means that a) students have shorter exposure time to their lecturer and hence may be challenged to provide accurate judgements and b) more course time needs to be spent on evaluations if all lecturers are to receive individual feedback, thus necessitating the availability of short and concise questionnaires. Lecturers may not be in charge of course organisation or the selection of the content taught. Accordingly, organisation- and content-based evaluation is not appropriate in this setting [2]. Most available questionnaires focussing on teaching quality and teacher characteristics are quite comprehensive, thus precluding their routine use. However, specific feedback on teaching quality for all lecturers involved in a course is needed for two reasons: First, improvements of teaching quality are much easier to achieve if lecturers can use reliable and truthful data to identify specific aspects of their teaching that can be improved. Second, as medical schools make increasing use of evaluation data to inform decisions on individual careers, reliable and valid evaluations are required for individual teachers in order to render these decisions as fair as possible.

Some English instruments addressing the specific characteristics of lectures in medical education exist, but published data mostly refer to presentations of isolated topics in one particular session [3, 4] or to applications in preclinical courses alone [4, 5]. In addition, none of the published evaluation tools is short enough to be used repeatedly during courses (i.e., for different lecturers). We are also not aware of questionnaires generating individual feedback for lecturers regarding criteria derived from the widely-used Stanford Faculty Development Program (SFDP) [6]. These criteria encompass the following seven interrelated factors: (1) learning climate, (2) control of the teaching session, (3) communication of educational goals, (4) promotion of understanding and retention of knowledge, (5) evaluation of the learner, (6) provision of feedback to the learner, and (7) promotion of self-directed learning [6].

The goal of this article is to investigate the underlying structure and the psychometric properties of a newly developed short German questionnaire for the evaluation of large-group lectures in medical education. In Study 1, we explored the underlying structure of the questionnaire and the appropriateness of the item response categories.

In Study 2, our intention was to confirm this structure in a second sample, as well as to assess the validity and reliability of the questionnaire. In addition, invariance of measurement of the questionnaire across different groups (i.e., medical schools and gender respectively) was investigated.

## Methods

### Description of the curricula

In the present investigation, two medical schools in Germany (at the University of Göttingen and the University of Hamburg) participated in the development and evaluation of a new instrument assessing lecturing performance in large groups based on the Stanford criteria mentioned above. However, the curricula of both medical schools differ to some extent. In Göttingen, a “traditional” curriculum is implemented, split up into a preclinical phase, followed by a phase of clinical practice. In contrast, Hamburg Medical School employs a reformed curriculum called “iMED”. The aim of this reformed curriculum is to integrate practical training in early stages of the educational programme. Consequently, there is no determination of preclinical/clinical sections.

### Item generation

Questionnaire development was based on selected and rephrased items from validated evaluation tools, matching the Stanford criteria. A pool of 190 items was generated from nine instruments in German and English language: the SEEQ (‘Students’ Evaluations of Educational Quality’) [7], the SFDP26-German (‘Stanford Faculty Development Program’) [8], the FESEM (‘Fragebogen zur Lehrveranstaltungsevaluation von Seminaren’ [Inventory for seminar evaluation]) [9], the UCEEM [10], the MedSEQ [11], the TRIL (‘Trierer Inventar zur Lehrveranstaltungsevaluation’ [Trier inventory for course evaluation]) [12], the MTEF-28 (‘Mayo Teaching Evaluation Form’) [13], and the SIR II (‘Student Instructional Report’) [14]. The questionnaires were chosen for multiple reasons: FESEM and TRIL were German questionnaires for lecturer evaluation and therefore chosen as item donors despite they were not specific for medical education. Only the factors *organisation, group interaction and workload/difficulty* of the SEEQ were selected for item donation. The other factors did not address areas relevant to the SFDP. From the SIR II the items of the factors *course organisation and planning, communication, student effort and involvement* were considered. The MedSEQ focused on the integration of the lecture in the curriculum. The MTEF28 was aligned to the SFDP categories and the UCEEM focused on learning atmosphere.

Selected items were rephrased and additional new items were drafted during the process. Relevance according to the Stanford criteria was discussed in a working group of 8 colleagues, consisting of 3 physicians, 3 psychologists, a sociologist and an education expert. If an item was in accordance with the SFDP-criteria and agreed upon as being highly relevant for medical education and clearly formulated, it qualified for retention. After revision according to the above- mentioned criteria including feasibility, 27 items were selected and, if only available in English, translated into German.

### Pilot testing and student involvement

In a consecutive pilot testing in the 2014 summer term, the 27-item version was handed out to 898 students at the medical schools of Göttingen and Hamburg. As the 27 - item version took too much time to be filled in several times during the term after teaching sessions a further reduction of the items was needed. In order to reduce item count, items with the lowest loading for the factors were deleted if there were several items assessing the corresponding SFDP category and if there was consensus in the working group, regarding relevance and clarity.

In cognitive debriefing sessions students were invited to discuss questionnaire instructions, items and answer scales with regard to content and wording in a formalized detailed way to avoid potential misinterpretations. Also, potentially missing questions were discussed; however, none were identified. A total of 30 students participated in this process, and some item wordings were revised according to their comments.

### Data collection

In winter term 2014/15, a total of 922 students evaluated teachers delivering large-group lectures at Göttingen or Hamburg Medical School (*Study 1*). Lecturers were eligible for inclusion if they taught the same group of students for at least 90 min. This was done to ensure a minimum level of exposure time to the same lecturer, and thus to increase comparability of individual judgements. In a course series, multiple lecturers could be included in the analyses if they met these criteria.

Following another round of discussions with subject matter experts and students during which two items were slightly rephrased, the final 14-item version was administered in *Study 2* (for included items please see Table 1; the original German questionnaire as well as verbatim translations of all items are provided in the Additional file 1 of this article). Throughout this manuscript, this final questionnaire version is referred to as SETMED-L (Student Evaluation of Teaching in MEDical Lectures). Data collection for Study 2 took place in summer term 2015 and winter term 2015/2016 at both medical schools. A total of 2740 student ratings were obtained. All items were rated on a five-point Likert scale (“strongly disagree”, “disagree”, “neither agree nor disagree”, “agree” and “strongly agree”).

**Table 1:** Descriptive analyses and factor solution for the modified Likert items with three categories

	Study 1 (*n* = 922)							Study 2 (*n* = 2740)							Factor solution	
Item	Mean (SD)	Median	Skew	Kurtosis	# imputed	Ceiling %	Floor %	Mean (SD)	Median	Skew	Kurtosis	# imputed	Ceiling %	Floor %	Cronbach’s alpha (if item removed)	Factor loadings
Factor 1: core teaching skills															0,892	
1) Session is well structured.	4,4 (0,81)	5	−1,3	1,44	7	55,1	0,4	4,3 (0,82)	4	-1,15	1,09	28	49,6	0,5	0,881	−0,493
2) Provided learning materials enhance understanding.	4,4 (0,79)	5	−1,45	2,23	8	56	0,7	4,3 (0,84)	4	−1,26	1,49	49	50	0,8	0,879	−0,619
3) Congruence between learning objectives and actual content.	4,3 (0,95)	5	−1,47	2,07	63	50,6	2,5	4,3 (0,87)	5	−1,54	2,66	128	51,3	1,8	0,884	−0,681
4) Teacher behaves respectfully towards students.	4,7 (0,59)	5	−2,88	10,76	18	79	0,6	4,8 (0,5)	5	−3	11,78	25	82,7	0,2	0,886	−0,855
5) Teacher comments students’ contributions and answers questions.	4,6 (0,74)	5	−2,04	4,99	49	67,7	0,9	4,6 (0,7)	5	−2,17	5,64	119	69,9	0,7	0,882	−0,698
6) Goal communication	3,8 (1,35)	4	−0,87	−0,49	53	43,2	10,4	4,2 (1,1)	5	−1,3	0,89	138	51,8	3,9	0,897	−0,689
7) Teacher enhances students’ interest in subject matter	3,9 (1,08)	4	−0,73	−0,33	131	38,1	2,2	3,8 (1,03)	4	−0,64	−0,25	42	31,6	2,2	0,877	−0,557
8) Teacher elucidates logical connections	4,3 (0,85)	4	−1,26	1,66	15	49,2	1,1	4,2 (0,85)	4	−0,93	0,52	48	41,6	0,4	0,875	−0,694
9) Use of examples relevant for practice	4,3 (0,92)	5	−1,36	1,36	20	56	1,2	4,2 (0,94)	4	−1,18	0,85	57	49,7	1,2	0,876	−0,798
10) Teacher expresses him−/herself clearly	4,5 (0,76)	5	−1,92	3,92	21	67,3	0,6	4,5 (0,77)	5	−1,8	3,64	51	62,5	0,8	0,879	−0,818
Factor 2: student activation skills															0,807	
11) Adequate balance between didactic teaching and student participation	3,7 (1,16)	4	−0,57	−0,55	13	30,7	4,8	3,6 (1,21)	4	−0,38	−0,88	62	28	5,3	NA^a^	0,677
12) Teacher asks questions to check student learning outcome	3,6 (1,24)	4	−0,53	−0,8	31	32,2	6,3	3,2 (1,27)	3	−0,11	−1,03	109	19,7	10,6	NA^a^	0,934
Factor 3: student workload															0,872	
13) Teaching pitched to the student level	4,4 (0,72)	5	−1,26	1,57	9	56	0,2	4,3 (0,76)	4	−1,14	1,4	21	48,3	0,3	NA^a^	0,916
14) Amount of content covered is appropriate	4,3 (0,82)	5	−1,24	1,34	6	52,4	0,6	4,2 (0,93)	4	−1,15	1,09	26	43,2	1,6	NA^a^	0,946

Oblimin rotation (only factor loadings ≥0.4 are reported). Original item wordings (German) and English translations can be found in the Additional file 1of this article

^a^ *NA* not available. For two item factors, Cronbach’s alpha if item removed could not be computed

In the 2015/2016 winter term, a subsample of *Study 2* (246 students at Göttingen University Medical Centre) completed the FEVOR questionnaire [9] in addition to the new instrument. To prove convergent validity, correlations with the FEVOR [9] were calculated. The FEVOR is a German lecturer-centred evaluation questionnaire for large groups, but not validated or developed for medical education. It consists of 20 items and five scales labelled PD (planning and presentation), US (student handling), IR (interestingness and relevance), SU (difficulty and workload) and VB (overall lecture evaluation). As the FEVOR focuses on similar characteristics (e.g. workload, student handling, interesting presentation), support of convergent validity of the newly developed SETMED-L questionnaire was expected.

Paper versions of the questionnaires were distributed to students and completed during the final 5 min of the lecture. All participating lecturers agreed to allocate this time for questionnaire completion.

### Data analysis

#### Missing values

Although raw data showed small proportions of missing values per item, a hot deck imputation was applied to increase the efficiency of estimates. Hot deck imputation refers to the method of replacing missing values for a non-respondent (called the recipient) with observed values from a respondent (called the donor) who is similar to the non-respondent regarding response patterns observed in both cases [15]. Replacement of missing data with observed data of the same dataset grants realistic values in spite of limited covariate information in our dataset. In the present investigation, the donor is selected randomly from a set of potential donors in the raw data set who have similar item response patterns as the recipients. After excluding a total of 48 unit non-response patterns from the raw data, a random hot deck imputation was performed on the 14 items with five Likert response categories within each sample (see Table 1 for the number of imputed values per item).

#### Item and scale descriptives and validity

To explore item distributions, mean, median, standard deviation, minimum, maximum, skewness, ceiling and floor effects were explored in both studies. Skewness values between −1 and 1 were considered acceptable [16]. Ceiling and floor effects were defined for items in which 20% or more of the item responses were in the last or first item category, respectively [17].

To inspect the appropriateness of the response scale, item characteristic curves (ICCs) were plotted. ICCs demonstrate the order of the probabilities to endorse the different response categories. Overlapping probability curves of two or more response categories indicate a violation of the assumption that the higher response category means a higher value on the latent factor. To obtain ICCs, all items were temporarily treated as if they scored on a single factor.

To examine reliability, internal consistency (Cronbach’s alphas) of the factors, found by exploratory factor analysis and tested by confirmatory factor analysis, was investigated.

Convergent validity was assessed by scale correlations with the FEVOR scales. Spearman’s r was used to compare similarities of the SETMED-L and the FEVOR. For all tests, significance level was set to α = .05.

#### Exploratory factor analysis

The underlying structure of the questionnaire was identified by performing an exploratory factor analysis on *Study 1* data. Since ceiling effects were to be expected [18], the common assumptions of independent and normally distributed residuals and continuous conditionally normal outcome in factor analysis are not fulfilled. An appropriate method for modelling this type of ordered-categorical item responses is the so-called full information item factor analysis (IFA). Within the framework of multidimensional item response theory (MIRT) methods, IFA appropriately handles the discrete nature of polytomous items (i.e., are items with more than two response categories) and exploits the information contained in the distinct item response vectors of the data set (i.e. not only the correlation matrix as in the traditional factor analysis approach) [19–21].

#### Confirmatory factor analysis

To investigate whether the factor structure obtained in *Study 1* could be reproduced, a confirmatory factor analysis (CFA) was performed in *Study 2*. For this analysis, the most appropriate factor structure obtained during the exploratory analyses in *Study 1* was used. Since CFA analyses with highly skewed items are associated with biased estimates [16], all items entered the CFA models as ordinal scaled variables with ordered response categories. Model parameters were estimated by weighted least squares means and variance adjusted estimator (WLSMV) [22].

The CFA was performed on both the original Likert items with five categories and the modified item responses with three categories. Model fit was assessed by robust estimates of the comparative fit index (CFI), the Tucker-Lewis index (TLI), and the root mean square error of approximation (RMSEA). Following the combinatorial rules of Hu and Bentler [23], the model fit is satisfactory if the model simultaneously satisfies the following cut-off points: CFI and TLI ≥ 0.96, and RMSEA <0.06.

#### Measurement invariance analyses

In the psychometric evaluation of questionnaires, measurement invariance (MI) analyses are performed in order to assess the extent to which a questionnaire measures identical constructs with the same factorial structure across relevant groups. A questionnaire showing measurement invariance has a broader applicability, given that the corresponding factor structure remains the same across groups. Thus, the comparison of factor scores across groups is based on the same metric and leads to valid conclusions. As student gender might also have an influence on the evaluation of teaching [24–26], measurement invariance should hold in order to compare the scores of female and male students. Research further suggests influence of the interaction between teacher and student gender [27–30].

MI analyses were performed on the combined samples of *Studies 1* and *2* to investigate whether the comparison of scores across the two medical schools, and between male and female students is based on the same factorial structure, thus yielding comparable results. CFA models were applied to study four types of measurement invariance: configural, loadings, intercepts and factor means invariance [31]. Configural invariance requires that the same factors and pattern of factor loadings are found in both groups. In addition loadings (or metric) invariance addresses the comparability of the factor loadings of each variable on each factor across groups. For intercepts (or scalar) invariance, the equality of the intercepts of the regression equations of the observed variables on the latent factors across groups is assessed. Finally, factor means invariance requires that the means of the latent factors are the same across groups.

As MI analyses test the factor model, the same indices (χ^2^, RMSEA, TLI, CFI) are used to assess the goodness of fit. The assessment of MI is based on Likelihood Ratio Tests (LRT), comparing the assumed invariant model (i.e. configural invariance) against the other types of invariance. Significance of the LRT means that the instrument is not equivalent across groups regarding the type of invariance being tested.

## Results

### Study 1

#### Descriptive statistics

For *Study 1*, data were collected in the 2014/2015 winter term. Of the 922 students, 385 (41.8%) were male, 484 (52.5%) were female and in 53 cases (5.7%) gender specification was not given. A total of 24 lecturers were evaluated (18 in Hamburg and 6 in Göttingen); 390 questionnaires were collected in Göttingen and 532 in Hamburg. Item means ranged between 3.69 and 4.74 and all items were negatively skewed, with 10 of them lying below the −1 threshold. For all items, more than 20% of the answers corresponded to the highest response category, thus representing a ceiling effect (see Table 1).

#### Exploratory factor analysis

Exploratory factor analysis started by assessing the appropriateness of the original Likert items with five categories. The corresponding item characteristic curves were plotted and it was observed that, for six items, the response categories were disordered, i.e. the probability of endorsing a higher category does not agree with higher scores on the latent construct level (curve overlapping). These results reflected the substantial ceiling effects observed in the original items. In order to improve the discriminative properties of the items, the original answer categories were re-coded as 1 (strongly disagree, disagree, neither agree nor disagree), 2 (agree), 3 (strongly agree). These modified Likert responses with three categories were re-analysed. The corresponding item characteristic showed a much better fit to the data than the original items with five answer categories.

#### Factor structure

Based on the results of the previous section, the factor structure of the questionnaire was investigated by using the modified Likert items with three categories. A total number of five factors was extracted from the IFA models. Subsequently, ANOVA tests were performed among those models, and the three-factor solution suggested by the scree plots of the parallel analysis was accepted (see Table 1. Further analyses are available from the first author on request).

According to their content, the three factors extracted were identified as *core teaching skills* (Factor 1), *student activation skills* (Factor 2), and *student workload* (Factor 3). The Cronbach’s alphas were between 0.81 and 0.89.

### Study 2

#### Descriptive statistics

Data for *Study 2* were collected in the 2015 summer term and the 2015/2016 winter term. In Göttingen, 2480 ratings of undergraduate medical students were collected, evaluating 43 lecturers. In Hamburg, 260 questionnaires rating 16 teachers were collected from second- and third-year students. Of all 2740 questionnaires, 789 (28.8%) were completed by male students, 1610 (58.8%) were completed by female students, and in 341 cases (12.4%) gender specification was missing. Item means ranged from 3.2 to 4.8 and all showed a negative skew with 10 items lying beyond the −1 threshold. For all items except one, more than 20% of the answers accumulated on the highest response category, indicating strong ceiling effects (see Table 1).

#### Confirmatory factor analysis (CFA)

The model fit indices of the CFA are reported in Table 2 for both the original Likert items with five categories and the modified items with three categories. A comparison of the fit indices of both models confirms that the modified Likert items with three categories outperform the model based on the original items with five categories (TFI 0.97 vs. 0.74, CFI 0.97 vs. 0.70, and RMSEA 0.08 vs. 0.16, respectively). Nonetheless, the best model fit was obtained by correlating the variances of items 5 and 9, and 7 and 8, respectively. Factor 1 showed a Cronbach’s alpha of 0.88, Factor 2 of 0.71 and Factor 3 of 0.79. Spearman’s interscale correlations (r_s_) between Factor 1 and the other two factors were r_s_(2697) = 0.62, *p* < .05 with Factor 2 and r_s_(2697) = 0.62, *p* < .05 for Factor 3. Factor 2 and 3 showed a Spearman’s interscale correlation of r_s_(2697) = 0.39, *p* < .05.

**Table 2:** Fit indices of the CFA models for the Likert items with three and five categories, respectively

Fit Index	Likert items with three categories	Likert items with five categories
CHISQ^a^	1467.27	9915.54
PVALUE^b^	< 0.001	< 0.001
CFI^c^	0.97	0.74
TLI^d^	0.96	0.70
RMSEA^e^	0.08	0.16
RMSEA CI LOWER^f^	0.06	0.25
RMSEA CI UPPER^g^	0.07	0.25

Items were treated as ordinal variables

^a^χ^2^ Test. ^b^ *p*-value of the χ^2^ Test. ^c^Comparative Fit Index: satisfying values should be >0.96 ^d^Tucker-Lewis Index: satisfying values should be >0.96. ^e^Root Mean Square Error Approximation: satisfying values should be <0.06. ^f^lower bound of RMSEA confidence interval. ^g^upper bound of RMSEA confidence interval

#### Measurement invariance analysis

##### Medical school location

The measurement invariance analysis reported in Table 3 revealed that the factor structure supported by the CFA models was similar for both medical schools. Even though the robust fit indices obtained for each type of measurement invariance are satisfactory regarding the cut-off-points (CFI, TLI, RMSEA) considered here, the LRT comparing the configural model to the assumed measurement invariance types suggests that some item responses do not behave equivalently across groups (see Table 3). Thus the questionnaire is not invariant across medical schools and group comparisons need to be interpreted with caution.

**Table 3:** Analysis for the CFA model with 3 factors and Likert items with 3 categories by study site and by gender

	Invariance analysis for CFA model by medical school				Invariance analysis for CFA model by gender			
Fit Index	Configural	Loadings	Intercepts	Means	Configural	Loadings	Intercepts	Means
CHISQ^a^	1856.2	1541.3	2351.1	2399.15	1712.76	1415.5	1719.03	1566.31
PVALUE^b^	< 0.001	< 0.001	< 0.001	< 0.001	< 0.001	< 0.001	< 0.001	< 0.001
CFI^c^	0.97	0.97	0.96	0.96	0.97	0.97	0.97	0.97
TLI^d^	0.96	0.97	0.96	0.96	0.96	0.97	0.97	0.97
RMSEA^e^	0.06	0.06	0.07	0.07	0.06	0.06	0.06	0.06
RMSEA CI LOWER^f^	0.06	0.06	0.06	0.07	0.06	0.06	0.05	0.06
RMSEA CI UPPER^g^	0.07	0.07	0.07	0.08	0.07	0.07	0.06	0.06
LRT TEST^h^	NA	0	0	0	NA	0.01	0.68	0.18
DF^i^	144	155	166	169	144	155	166	169

^a^χ^2^ Test statistics ^b^ *p*-value of the χ^2^ Test ^c^Comparative Fit Index: satisfying values should be >0.96 ^d^Tucker-Lewis Index: satisfying values should be >0.96 ^e^Root Mean Squared Error Approximation: satisfying values should be <0.06 ^f^lower bound of the RMSEA confidence interval ^g^upper bound of the RMSEA confidence interval ^h^Likelihood ratio test (LRT) of the configural model vs. the other types of measurement invariance models ^i^Degrees of Freedom

##### Gender

The results of the measurement invariance analyses by gender reported in Table 3 suggest satisfactory robust indices for all measurement invariance models. In addition, the LRT statistics support the notion of measurement invariance by gender, except for the factor loadings invariance. Thus the comparison of scores between male and female students is based on a similar factor structure based on the same latent scale.

#### Convergent validity

Spearman correlations (r_s_) of the three SETMED-L factors with the FEVOR scales ranged from −0.6 to 0.64. Factor 1 (core teaching skills) demonstrated high correlations with PD (planning and presentation, r_s_ = 0.64(231), *p* < .05) and IR (interestingness and relevance, r_s_ = 0.6(231), *p* < .05). Factor 2 (student activation) indicated lower correlations with IR (r_s_ = 0.48(231), *p* < .05) and PD (r_s_ = 0.31(231), *p* < .05). Factor 3 (student workload) also correlated with IR (r_s_ = 0.44(229), *p* < .05) and PD (r_s_ = 0.39(229), *p* < .05) on a lower level. FEVOR’s scale for global evaluation (VB) showed moderate negative correlations with all SETMED-L factors (F1: r_s_ = −0.6(225), *p* < .05; F2; r_s_ = −0.41(225), *p* < .05; F3: r_s_ = −0.57(223), *p* < .05). The scale for student handling (FEVOR’s US) yielded small but significant correlations with the SETMED-L factors (F1: r_s_ = 0.34(231), *p* < .05; F2: r_s_ = 0.25(231), *p* < .05; F3: r_s_ = 0.23(229), *p* < .05). No significant correlations were found for the FEVOR factor SU (difficulty and workload) with any SETMED-L factors (F1: r_s_ = −0.06(223), *p* = .38; F2: r_s_ = 0.03(223), *p* = .68; F3: r_s_ = −0.004(221), *p* = .953).

## Discussion

The development of the 14-item evaluation questionnaire of teacher performance in large-group lectures – the SETMED-L – was based on the seven Stanford criteria for good teaching; the majority of existing evaluation forms for this purpose are not based on this or a similar framework. Psychometric properties were investigated in two consecutive studies. The exploratory analyses in *Study 1* resulted in three factors: core teaching skills (10 items), student activation skills (two items), and student workload (two items; see Table 1). In addition, analysis of the item characteristic curves indicated that the original Likert items with five categories should be recoded to Likert items with three categories in order to enhance the discriminating properties of individual items. Cronbach’s alpha of the three hypothesized scales ranged between 0.81 and 0.89, indicating a good internal consistency.

In *Study 2*, the three-factor solution was tested in several CFA models based on the modified three Likert categories. The fit indices of the hypothesized three-factor model were satisfactory. All factors showed a good internal consistency, with Cronbach’s alphas ranging between 0.71 and 0.88. Measurement invariance analyses concerning the performance of the questionnaire in both medical schools revealed large measurement biases. Given that both medical schools implemented different types of curricula, the differences may be accounted for by the overall curriculum structure and the students’ differing appreciation of it. In contrast, the measurement invariance analyses for gender did not yield significant differences. Consequently, scores obtained from male and female students are comparable.

The convergent validity analyses with the FEVOR [9] showed mixed results. Factor 1 (core teaching skills) demonstrated Spearman correlations ≥0.5 with the FEVOR scales PD (planning and presentation) and IR (interestingness and relevance). Both Factor 2 (student activation) and Factor 3 (student workload) were significantly correlated with IR, but correlations were weaker. The SETMED-L scales seemed to capture related but nonetheless differing constructs from those which the FEVOR scales do. Questions about student activation skills are unique to the SETMED-L. As activation might lead to increased interest in lecture content, the correlation with IR seemed reasonable; also the positive association of interestingness/relevance and workload seemed useful.

A major interesting point was the non-significant correlation between Factor 3 (student workload) and FEVOR’s SU (difficulty and workload), as technically they should have measured a similar latent construct. This unexpected result may be due to different item response categories. While on the FEVOR questionnaire, students are required to quantify the workload between “too little” and “too much”, the items of the SETMED-L asked students to rate the appropriateness of the workload between “strongly disagree” and “strongly agree”, and thus they were not discriminating the directions of inappropriateness explicitly.

### Practical implications for evaluation in medical education

To our knowledge, the SETMED-L is currently the shortest questionnaire evaluating teacher performance in medical lectures. In addition to its items being derived from a widely-used framework of high-quality teaching, its psychometric properties are favourable, and the analyses regarding measurement invariance suggest that student gender does not affect evaluation results. In addition, few instruments apart from the SETMED-L include items of student activation. Due to these advantages over existing questionnaires, the SETMED-L lends itself to routine use in courses with multiple lecturers. Curriculum-wide implementation of the tool will provide a rich database from which teacher rankings may be derived and these evaluations may result in informed decisions on individual career pathways.

Apart from this summative function, one major purpose of the SETMED-L is to provide formative feedback to teachers thriving to further improve their didactic skills. Information on specific aspects of teaching can be used to tackle corresponding areas of potential improvement. From a faculty development perspective, lecturers who have received their individual evaluation results could be invited to participate in teacher trainings tailored to their specific needs, and repeated measurements following training could be used to assess the progress made. Qualitative studies assessing teacher and student perceptions of the new tool following curriculum-wide implementation are currently under way [32].

### Limitations

There are several limitations to our study. Firstly, the participation of teachers and students was voluntary, which may have caused a selection bias favouring more motivated teachers and students. To some extent, this may explain the large ceiling effects observed. There also may exist a self-selection bias among students, since large-lecture attendance is voluntary at both medical schools. Hence, critical or dissatisfied students might not even have attended the lecture. For ethical reasons, we did not collect any personal data except student gender. In Study 1, information on gender was missing in 5.7% of cases. The observed distribution (41.8% male; 52.5% female students) largely reflects current enrolment data at both medical schools involved in this study. A recent national survey including almost 20,000 German medical students yielded rates of 35% (male) vs. 65% (female) [33].

Secondly, student ratings of teacher performance may have been influenced by halo effects; i.e. when students’ evaluations of a lecturer’s performance is influenced by the sympathy felt towards the lecturer [34]. One way to reduce these would be to train students how to use the questionnaire and its scales [35]. Thirdly, the large ceiling effects may also have been caused by items not adequately capturing the whole range of lecturer behaviour along the three dimensions of teaching skills. Our results indicate that Likert items with three categories were better suited to identify differences between lecturers. Due to the fact that data were anonymised and no personal data were collected, additional validation analysis involving factors such as student performance, socioeconomic background, etc. was not feasible. It should be noted that all results reported in the manuscript – including validity outcomes – were derived from the German version of the questionnaire. Items were translated for presentation purposes only. Validation of an English version of the questionnaire would be a prerequisite for transferring our current findings to the translated version provided in the Additional file 1.

### Suggestions for future research

Ceiling effects were a major problem in psychometric testing. Model fit was improved by merging the original response categories into three categories. Future research may aim to investigate whether a three-point Likert scale, dichotomous items, or formulating larger differences between categories would be able to minimise ceiling effects in the responses. Additional items may help discriminate in the upper scores but at the cost of increasing total item count and therefore completion time. Furthermore, the specific mechanisms by which measurement bias is present across medical schools should be further investigated. Moreover, future research may explore specific curricular elements enhancing the quality of large lecture groups in medical education. Finally, the effects of evaluation training for students on halo effects should be investigated.

## Conclusion

The newly developed SETMED-L questionnaire represents a reliable, valid, short and yet comprehensive evaluation tool of teaching performance in medical lectures based on the seven Stanford criteria. Three factors capturing teaching skills, student activation skills, and student workload were identified. The measurement invariance analyses suggested that differences in both medical schools may play a significant role in the students’ perceptions of teaching performance. Comparisons across medical schools should be applied with caution. However, the SETMED-L did not show signs of measurement bias regarding male and female students, thus allowing gender comparisons of scale scores. The questionnaire can be used in large lecture series that involve multiple lecturers. The short length allows multiple evaluations during the term with acceptable loss in lecturing time (approximately 5 min per use). Results can be used to inform individual lecturers about their performance or course organisers about the overall outcome of the lecture series, thus granting viable information for individual and curricular improvement.

## Additional file

Additional file 1: Original German questionnaire and English translation. (PDF 134 kb)

## Abbreviations

ANOVA: Analysis of variance
CFA: Confirmatory factor analysis
CFI: Comparative fit index
ICC: Item characteristic curves
IFA: Item factor analysis
LRT: Likelihood ratio test
MI: Measurement invariance
MIRT: Multidimensional item response theory
RMSEA: Root mean square error of approximation
SFDP: Stanford faculty development program
TLI: Tucker-Lewis index
WLSMV: Weighted least squares means and variance
