# Supplementary material for: Evaluation of large-group lectures in medicine – development of the SETMED-L (Student Evaluation of Teaching in MEDical Lectures) questionnaire
Source: BMC Med Educ. 2017 Aug 18;17:137. doi: 10.1186/s12909-017-0970-8 (PMC5563045; doi:10.1186/s12909-017-0970-8)
Supplement: Additional file 1: — Original German questionnaire and English translation. (PDF 134 kb) [file 12909_2017_970_MOESM1_ESM.pdf]

Additional file 1: Original German questionnaire and English translation (see p. 3)

Universitätsmedizin Göttingen	BMBF-Projekt "EAGLE"<br>SS 2015	UMG

Markieren Sie so: ☐ ☒ ☐ ☐ ☐ Bitte verwenden Sie einen Kugelschreiber oder nicht zu starken Filzstift. Dieser Fragebogen wird maschinell erfasst.

Korrektur: ☐ ■ ☐ ☒ ☐ Bitte beachten Sie im Interesse einer optimalen Datenerfassung die links gegebenen Hinweise beim Ausfüllen.

Liebe Studierende,
bitte beurteilen Sie die von Ihnen besuchte Lehrveranstaltung anhand der folgenden Aussagen. Denken Sie dabei an alle Vorlesungstermine bei diesem Dozenten bzw. dieser Dozentin innerhalb des Moduls, an dem Sie gerade teilnehmen.
Bitte lesen Sie jede Aussage aufmerksam durch und beurteilen Sie, wie sehr Sie dieser Aussage zustimmen.
Mit der sorgfältigen Beantwortung dieses Fragebogens unterstützen Sie gute Lehre.
Bitte machen Sie pro Aussage nur ein Kreuz in das Kästchen, dessen Antwort am ehesten auf Sie zutrifft.

1. Personenbezogene Daten

1.1 Bitte geben Sie Ihr Geschlecht an: ☐ männlich ☐ weiblich

2. Fragen zur Lehrveranstaltung

		trifft gar nicht zu	trifft wenig zu	trifft teilweise zu	trifft ziemlich zu	trifft völlig zu
2.1	Der Schwierigkeitsgrad der Veranstaltung ist an den Wissensstand der Studierenden angepasst.	☐	☐	☐	☐	☐
2.2	Der in der Veranstaltung bearbeitete Stoffumfang ist angemessen.	☐	☐	☐	☐	☐
2.3	Die Veranstaltung ist klar gegliedert.	☐	☐	☐	☐	☐
2.4	Die vom Dozenten/von der Dozentin während der Veranstaltung eingesetzten Materialien und Medien (z.B. Folien, Präsentation, Tafel, Film, Präparate, Arbeitsblätter etc.) sind hilfreich für mein Verständnis.	☐	☐	☐	☐	☐
2.5	Die in der Veranstaltung behandelten Inhalte stimmen mit den zu Beginn dargestellten Lernzielen überein.	☐	☐	☐	☐	☐
2.6	Die Veranstaltung beinhaltet eine gute Mischung aus Wissensvermittlung durch den Dozenten/die Dozentin und aktiver Beteiligung der Studierenden.	☐	☐	☐	☐	☐
	**Der/Die Dozent/in**					
2.7	- verhält sich den Studierenden gegenüber respektvoll.	☐	☐	☐	☐	☐
2.8	- geht auf Fragen und Beiträge der Studierenden ausreichend ein.	☐	☐	☐	☐	☐
2.9	- stellt die zentralen Inhalte (konkrete Lernziele) zu Beginn der Veranstaltung dar.	☐	☐	☐	☐	☐

2. Fragen zur Lehrveranstaltung [Fortsetzung]

**Der/Die Dozent/in**	trifft gar nicht zu	trifft wenig zu	trifft teilweise zu	trifft ziemlich zu	trifft völlig zu
2.10 - stellt Verständnisfragen, um die gelernten Inhalte abzusichern.	☐	☐	☐	☐	☐
2.11 - gestaltet die Veranstaltung so, dass mein Interesse am Themengebiet gefördert wird.	☐	☐	☐	☐	☐
2.12 - macht Zusammenhänge deutlich.	☐	☐	☐	☐	☐
2.13 - veranschaulicht die Inhalte anhand von anwendungsbezogenen/ praxisnahen Beispielen.	☐	☐	☐	☐	☐
2.14 - drückt sich verständlich aus.	☐	☐	☐	☐	☐

German wording	English translation	Item wording in Table 1
Bitte geben Sie Ihr Geschlecht an.	Please indicate your sex.	
Der Schwierigkeitsgrad der Veranstaltung ist an den Wissensstand der Studierenden angepasst.	The difficulty level of the session is pitched to the student level.	Teaching pitched to the student level
Der in der Veranstaltung bearbeitete Stoffumfang ist angemessen.	The amount of content covered in the session is appropriate.	Amount of content covered is appropriate
Die Veranstaltung ist klar gegliedert.	The session is well-structured.	Session is well-structured
Die vom Dozenten/von der Dozentin während der Veranstaltung eingesetzten Materialien und Medien (z.B. Folien, Präsentation, Tafel, Film, Präparate, Arbeitsblätter etc.) sind hilfreich für mein Verständnis.	The ressources used by the teacher during the session (e.g., slides, presentations, blackboard, video, specimen, worksheets etc.) enhance my understanding.	Provided learning materials enhance understanding
Die in der Veranstaltung behandelten Inhalte stimmen mit den zu Beginn dargestellten Lernzielen überein.	The content covered is aligned to the learning objectives outlined at the beginning of the session.	Congruence between learning objectives and actual content
Die Veranstaltung beinhaltet eine gute Mischung aus Wissensvermittlung durch den Dozenten/die Dozentin und aktiver Beteiligung der Studierenden.	The session comprises a good balance of didactic teaching and student participation.	Adequate balance between didactic teaching and student participation
**Der/Die Dozent/in…**	**The teacher…**	
...verhält sich den Studierenden gegenüber respe	…behaves respectfully towards students.	Teacher behaves respectfully towards students
...geht auf Fragen und Beiträge der Studierenden ausreichend ein.	…responds adequately to students' questions and	Teacher comments students' contributions and answers questions
...stellt die zentralen Inhalte (konkrete Lernziele) zu Beginn der Veranstaltung dar.	…outlines central learning objectives at the beginning of the session.	Goal communication
...stellt Verständnisfragen, um die gelernten Inhalte abzusichern.	...asks questions to check student learning outcome	Teacher asks questions to check student learning outcome
...gestaltet die Veranstaltung so, dass mein Interesse am Themengebiet gefördert wird.	…designs the session in such a way that my interest in the subject matter is enhanced.	Teacher enhances students' interest in subject matter
...macht Zusammenhänge deutlich.	…elucidates logical connections.	Teacher elucidates logical connections
...veranschaulicht die Inhalte anhand von anwendungsbezogenen/ praxisnahen Beispielen.	...uses realistic examples to illustrate teaching content.	Use of examples relevant for practice
...drückt sich verständlich aus	…expresses him-/herself clearly.	Teacher expresses him-/herself clearly
